# Monitoring butterflies using counts of puddling males: A case study of the Rajah Brooke's Birdwing (*Trogonoptera brookiana albescens*)

**DOI:** 10.1371/journal.pone.0189450

**Published:** 2017-12-12

**Authors:** Chooi-Khim Phon, Laurence G. Kirton, Norma-Rashid Yusoff

**Affiliations:** 1 Tropical Forest Biodiversity Centre, Forest Research Institute Malaysia (FRIM), Kepong, Selangor, Malaysia; 2 Zoological and Ecological Research Network, Institute of Biological Sciences, Faculty of Science, University of Malaya, Kuala Lumpur, Malaysia; INRA-UPMC, FRANCE

## Abstract

The Rajah Brooke's Birdwing, *Trogonoptera brookiana*, is a large, iconic butterfly that is facing heavy commercial exploitation and habitat loss. Males of some subspecies exhibit puddling behavior. A method of conservation monitoring was developed for subspecies *albescens* in Ulu Geroh, Peninsular Malaysia, where the males consistently puddle in single-species aggregations at stable geothermal springs, reaching well over 300 individuals when the population is at its highest. Digital photography was used to conduct counts of numbers of males puddling. The numbers of birdwings puddling were significantly correlated with counts of birdwings in flight, but were much higher. The numbers puddling during the peak hour were correlated with numbers puddling throughout the day and could be predicted using the numbers puddling at an alternative hour, enabling flexibility in the time of counts. Average counts for three images taken at each puddle at three peak hours between 1400–1600 hours over 2–3 days were used as a monthly population index. The numbers puddling were positively associated with higher relative humidity and brightness during monitoring hours. Monthly counts of birdwings from monitoring of puddles over a period of two years are presented. The minimum effort required for a monitoring program using counts of puddling males is discussed, as well as the potential of using the method to monitor other species of puddling butterflies.

## Introduction

**Fig 1:**
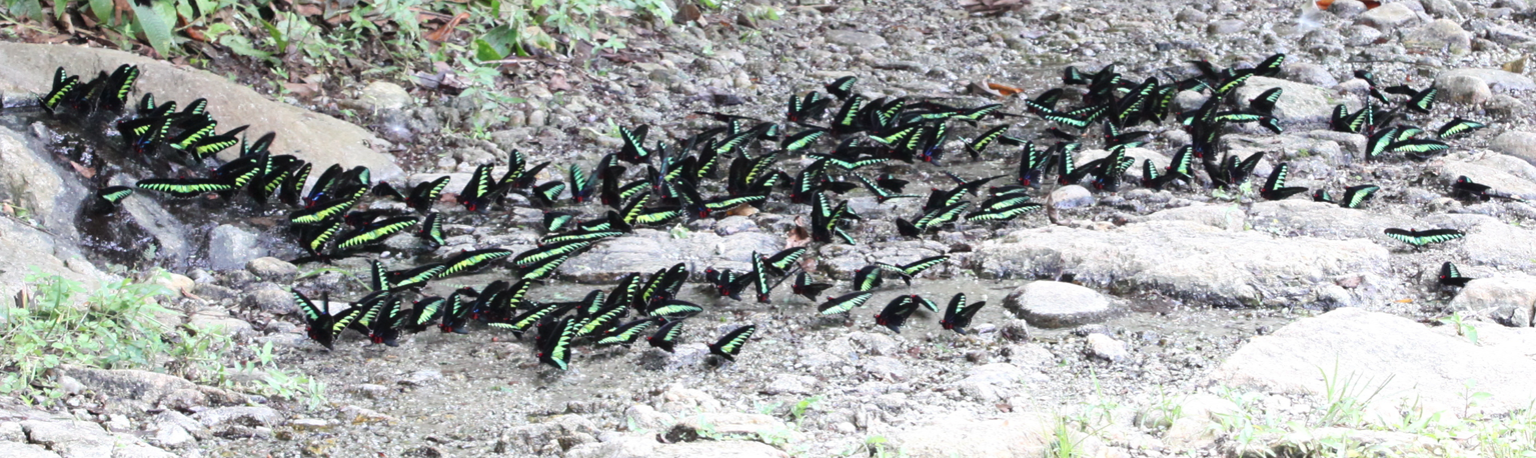
Puddling males of the Rajah Brooke’s Birdwings in Ulu Geroh.

The Rajah Brooke’s Birdwing, *Trogonoptera brookiana* Wallace (Lepidoptera, Papilionidae) is a large black and metallic green butterfly that has become a national icon in Malaysia. In Peninsular Malaysia, it occurs as two subspecies. Subspecies *albescens* Rothschild is confined to the western side of the central mountain range from the state of Perak southward to Negeri Sembilan, and subspecies *mollumar* D’Abrera is confined to the eastern plains of Johor and southeast Pahang, with small populations occurring further northeast in Terengganu [1, 2]. Its host plant is *Aristolochia foveolata* Merr., a climbing plant that is said to have a scattered distribution in primary lowland and hill forests up to an altitude of 500 m, and has an IUCN conservation status of near threatened in Peninsular Malaysia [3] based on the adapted Malaysian IUCN criteria [4]. The males of *T*. *b*. *albescens* are unusual among birdwings for their communal puddling behavior along river banks [1], usually in single-species groups that may occasionally reach hundreds of individuals (Fig 1). The precise reasons for this behavior have not been investigated, but studies indicate that some male butterflies concentrate salts and transfer them to females in the spermatophores as a nuptial gift that can enhance fecundity and longevity [5–8]. In *T*. *brookiana*, stable aggregations appear to be associated with hot springs or geothermal seepages [1], while males may briefly alight to puddle at other locations along rivers and forest roads, or for longer periods where there is a transient source of salts such as urine [1]. In Malaysia, as in other non-volcanic regions, hot springs result from the heating of water by granitic intrusions of cooling magma or from the heating of cold water that seeps deep into the earth’s mantle through faults and rises to the surface by convectional pressure [9]. The reasons for the attraction of *T*. *brookiana* to certain hot springs have not been investigated, but could be related to thermoregulation or mineral and organic content in the water, or a combination of these reasons.

Despite being protected under Act 76 [10] and Act 716 [11], which allow for collection and trade with a license, and listed in Appendix II of CITES [12], which allows for trade under a permit, the species still faces heavy collecting pressure and a large volume of trade. Between 1987 and 2005, the total reported export trade for this species in Malaysia was 22,091 individuals, with most specimens collected from the wild [13]. Between 2001 and 2010, the total reported export trade of wild specimens was 5,060 individuals [14].

Although there has not been any prior systematic population monitoring, there is a general public perception that numbers of this birdwing have declined over the years. Reasons for a decline in the population could be over-collecting, but more so logging and conversion of forests for agriculture and human habitation. A long-term monitoring program is needed to help understand seasonal variations and longer term population changes. The information gained from such a monitoring program would support conservation efforts [15]. In recent years, ecotourism has been a driving force for conservation efforts for this butterfly in some sites. Notably, in the Malaysian village of Ulu Geroh, the site of this study, indigenous Semai guides promote birdwing watching as one of the main ecotourism activities [16].

Before a monitoring program can be implemented, it is essential to develop a monitoring method suitable for the target species. Detectability is a major concern [17]. The transect walk method, pioneered by Pollard [18], is often used in butterfly monitoring, especially in European countries. However, this birdwing is infrequently encountered in flight in the forest away from its puddling sites. Therefore, transects would need to be long, and would require extended sampling times to yield even small counts. Where the birdwing puddles, counts in flight can become difficult because of high numbers in a single location, and confusion could arise from multiple counts of the same individual in flight. In several capture-mark-release-recapture trials that we conducted in the field, released birdwings changed their flight paths, and there were no recaptures. It has been shown in some species that handling butterflies to mark them causes avoidance [19], or reduces recaptures in comparison to marking without handling [20, 21]. Counting immature stages on naturally occurring hostplants as described by Lindzey and Connor [22] for the Mission Blue butterfly cannot be applied in this case because the host plant is difficult to find and is an inaccessible climbing vine. Monitoring caterpillars on planted vines, such as has been done for the Richmond Birdwing in Australia since 1992 [23], is also not possible because the host plant is difficult to propagate. Bait traps, which have been used, for example, by Pozo et al. [24] to monitor some fruit feeding species, are also not a viable option in this case as the species is not attracted to bait. However, the puddling behavior of male *T*. *b*. *albescens* provides potential for counts of puddling birdwings to be used to monitor populations, given that the number of males should be proportional to the number of females. Little is known of the attractive range of the puddles or the behavioral factors that influence congregation. Unlike fruit baits, the degree of sensory detection from a distance is likely to be small. However, males of *T*. *b*. *albescens* have a habit of flying distances along river banks [25] in what is likely to be patrolling behavior that has been described for other butterflies [26–28]. They may fly low and alight momentarily on the banks of rivers. Sensory perception of thermal and chemical cues at puddles may be involved at a close range. They may also be attracted visually to the presence of other congregating males, and our observations at Ulu Geroh suggest they may linger for days in the vicinity of puddles, indicating a learned behavior.

In this study, we test the use of counts of puddling Rajah Brooke’s Birdwings as a method of monitoring a local population using an index of abundance, and discuss its suitability and limitations. We determine for the test site the optimum duration of a monitoring session, the best times of day and the effects of weather. In addition, the reliability of the method was tested against a modified transect count. Monthly data are presented for a two-year monitoring period.

## Materials and methods

The study was conducted in one of the few remaining sites where relatively large numbers of male Rajah Brooke’s Birdwings consistently puddle in single-species groups. The puddling occurs just above a river (Sungai Geroh) at the forest fringe beside the very small indigenous-community village of Ulu Geroh (4° 26' 24.9" N, 101° 15' 01.8" E) in the Malaysian state of Perak. The village is planted with fruit trees and flowering bushes interspersed among small wooden village houses, and is closely flanked by the extensive Bukit Kinta Forest Reserve. Preliminary surveys identified two main puddling sites about 30 m apart that formed the basis for the study (Fig 1). A small puddling group occurred next to the larger of the two puddling groups in the first three months of the study and was included. The main puddle is a stable resource as it is a small hot spring. Consequently, it is consistently warmer to touch than other adjacent moist areas. Very small occasional puddling groups that were scattered much further away were excluded.

Visual field counts are impractical and unreliable when large numbers of butterflies puddle. Therefore, we used digital photography to aid in obtaining counts. Two cameras, a Canon EOS 5D Mark II with an EF 70–200 mm f / 2.8 L USM lens, and a Nikon Coolpix P5100, were mounted on tripods 5–10 m from the two main puddling sites and used to capture images of puddling birdwings. The EOS 5D Mark II, which captures images of higher resolution, was used for the larger group. The distance of the cameras and their orientation were adjusted each day and month to frame all the birdwings in each large puddling group and to obtain the best view. Two operators communicated using walkie-talkies and triggered the cameras simultaneously at the different puddles. The numbers of birdwings on the small transient puddle were small and were counted visually. Counts of birdwings in the images were made with the aid of the image analysis software, ImageJ 1.44p.

### Comparison of transect counts and puddling counts

A comparison of the technique was made with transect counts that were conducted near the puddling sites. Male birdwings in flight patrolling the river were counted along a 180×20 m belt transect by the same observer walking through once at a slow and steady pace for the first 15–20 minutes of each hour from 0900–1700 hrs. The count zone was the full 20-meter width of the transect, up to the height of the tree canopies, and with a forward view as far as the eye could see. The location and direction of the transect walk was such that the observer could avoid disturbing the two puddling sites. It began about 20 m downstream of the largest puddle and moved further downstream parallel and adjacent to the river, which was up to about 20 m wide. This stretch of the river edge encompassed by the belt transect was observed to have the most birdwings in flight, and the birdwings were sufficiently large and distinctive to be recognized easily within the belt. Back and forth flights of what was recognizably the same individual birdwing circling within or across the belt transect were counted as a single sighting. Transect and puddling counts were obtained for three high (January to March 2010) and three low (September, October and December 2010) population months for three days each month but, due to logistical limitations, two days in January 2010. Three photographs were taken one minute apart at the start of every hour from 0900–1700 hrs. The counts of puddling birdwings in the three images at the start of every hour were averaged to obtain hourly averages. Hourly averages of birdwings on puddles and hourly counts of birdwings in flight were averaged to obtain daily averages which were averaged to obtain the monthly averages. Pearson’s correlation was used to examine the relationship between the monthly average numbers of birdwings puddling and in flight.

### Sample requirements for puddling counts

To determine the sample requirements for counts of puddling birdwings, a few component studies were undertaken. In the first component study, the optimum number of consecutive images that was needed to obtain a reliable count of puddling birdwings at any given hour was determined from two sets of images taken on a single day, beginning 1100 hrs and 1500 hrs. Each set comprised 30 images taken a minute apart, with no disturbances to the birdwings. The 30-image sets were divided into six consecutive blocks of five images each. The averages for the first image, the first three images, and all five images in each block were taken, and the averages of the six blocks were compared for increasing numbers of images. Their degree of similarity was examined using 95% confidence intervals. Based on the results (described in the results section), the average of three images at the start of each hour was used for all subsequent tests. However, if there was a disruption at the start of an hour, such as a sudden gust of wind that caused the puddling birdwings to fly, the three images were taken 10 minutes later. The remaining three component studies utilized the same 6-month puddling dataset that was used in the comparison between transect counts and puddling counts. The second component study examined the number of monitoring hours needed. To determine whether counts at a single hour would be representative of counts throughout the day, the numbers of birdwings puddling at 1600 hrs were correlated against the average numbers of birdwings puddling from 0900–1600 hrs. Counts over a two- or three-day period each month were first averaged for these time periods. The choice of 1600 hrs was based on an observed peak puddling period from 1400–1600 hrs. A third study analyzed whether the numbers of birdwings puddling at a chosen time of day in a monitoring program can be predicted from counts taken at an alternative time of day. This would be important if, for example, rain, heavy cloud or circumstances prevent the count being made at the chosen monitoring period. Though strictly speaking not intended for bivariate data, regression was used to analyze the relationship between the numbers of birdwings puddling at 1100 hrs and 1600 hrs, with the numbers at 1100 hrs taken as an independent variable in the regression analysis to enable a predicted estimate of the number at 1600 hrs. A fourth component study analyzed the number of monitoring days required for a representative count. This was determined by inspecting the correlation matrix for the average counts obtained on the first, second and third day at 1600 hrs. For the single month with only two monitoring days, the third day’s count was treated as a missing value in the correlation.

### Application of the technique

Monitoring was carried out for a period of two years from January 2010 to December 2011 using a protocol refined from the above-mentioned studies. Three images were captured a minute apart beginning on the hour from 1400 to 1600 hrs for three days each month, except for the first month and last three months in which two days were used due to logistical limitations. The monitoring days were consecutive except on one month in which the three sampling days occurred over a four-day period due to a day of rain. Environmental variables were recorded or scored 30 minutes into each monitoring hour and averaged for each day. Temperature and relative humidity were recorded with a single reading at each hour using a calibrated datalogger (Blue Gizmo BG-DL-01). Brightness and rainfall were scored hourly on a scale of 1–3 (dull, moderately bright, and very bright) and 0–2 (dry, drizzle, and light rain), respectively. Monitoring was not carried out if there was heavy rain during the actual period of monitoring. In addition to hourly scores, a separate score was given each day to generalize brightness and rainfall from early morning to late evening (i.e. all-day brightness and all-day rainfall), using the same scales as the hourly scores. The data was standardized using Z-scores, and analyzed using a General Linear Model (GLM) in Minitab 17^®^ to determine whether environmental variables affected counts of puddling birdwings. Z-scores were used to obtain cross-comparable regression coefficients for the environmental variables. Daily average counts were the response variable in the GLM, and month was treated as a fixed variable, with days nested within months. Daily environmental measurements and scores were treated as covariates. A full analysis was conducted with all variables, and then stepwise variable selection with an α to enter and α to remove of 0.15 was used to obtain a final reduced model.

## Results

### Comparison of transect counts and puddling counts

**Fig 2:**
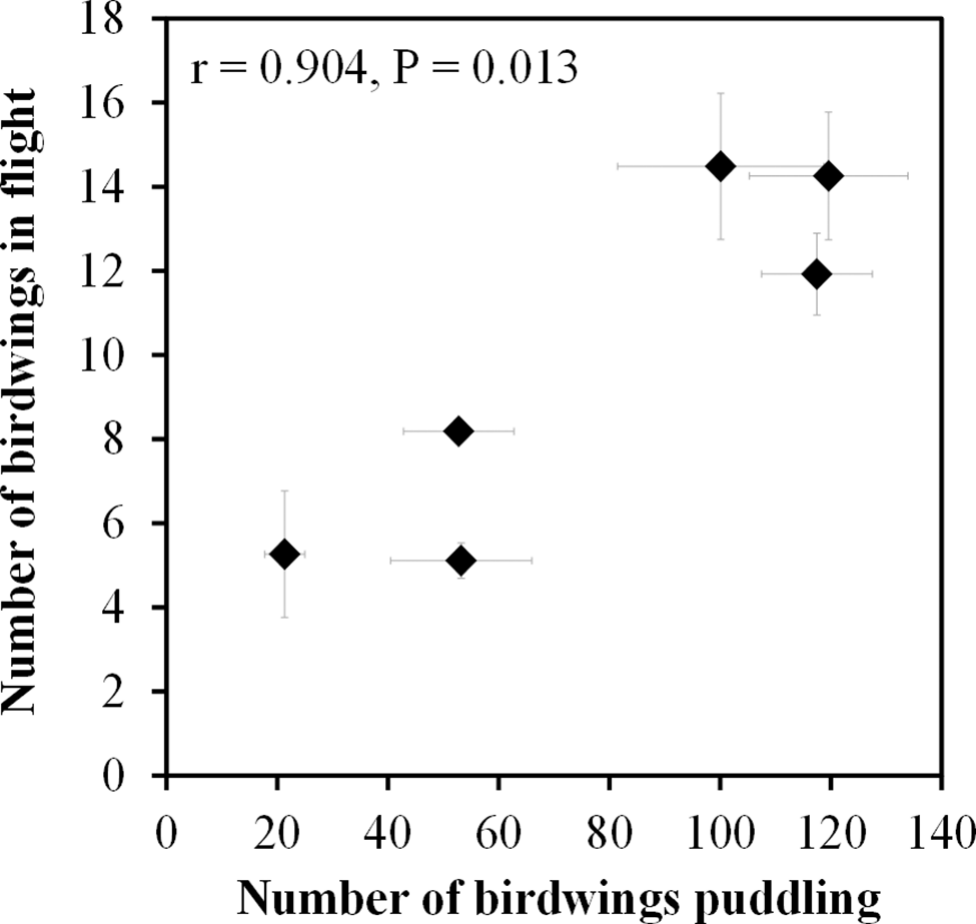
Correlation of numbers of birdwings in flight and numbers puddling. Data shown are monthly means for 6 monitoring months, with standard error bars.

Computer-assisted counts of large puddling birdwings in the digital images from both types of cameras, and manual field counts of small numbers of puddling birdwings, were all equally manageable and accurate because of the large size of the birdwings, yielding actual numbers rather than estimates. Many birdwings were observed flying out from the trees near the puddling sites in the early morning and flying back to rest on these trees in the evening. Occasionally, when there were disturbances, such as people passing by, birds landing nearby, a leaf fall, and gusts of wind, puddling birdwings became unsettled and flew away, but remained nearby and eventually came back to rest at the puddle. In one such case, when birds flew near to the puddle, all but five of the 20 puddling birdwings flew away and landed on trees nearby, but they returned to their original numbers at the puddle after 10 minutes (S1 Fig). Recovery times were similar when other disruptive events occurred. No disruptions occurred during the comparison between transect counts and puddling counts. There was a significant, positive correlation (Pearson’s correlation coefficient, r = 0.904, P = 0.013) between the monthly average numbers of birdwings in flight and the numbers of birdwings puddling from 0900–1700 hrs (Fig 2). Average counts of birdwings in flight numbered only about 5–14 individuals, whereas counts of puddling birdwings were much higher, between 18 and 120 individuals. The ratios of birdwings at puddles to birdwings in flight in the high season monthly counts were in the range of 6.9–9.8 and averaged 8.4. Ratios during the low season had a range of 3.5–10.4, with an average of 7.0.

### Sample requirements for puddling counts

**Fig 3:**
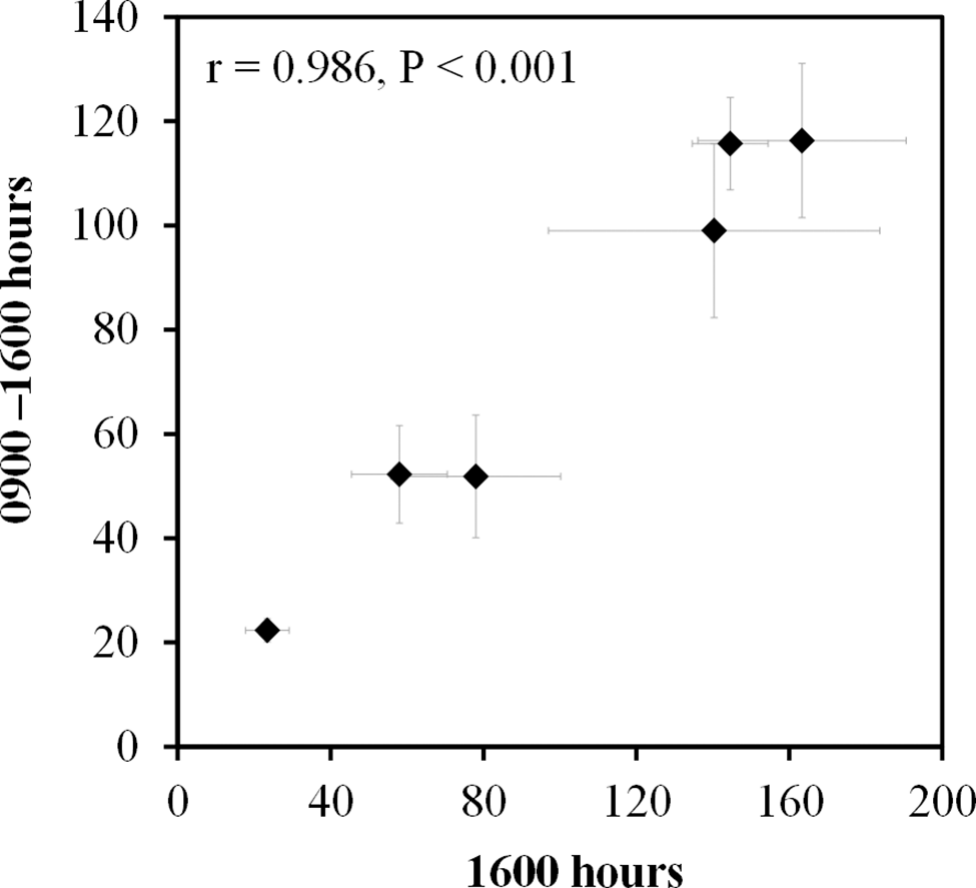
Relationship between numbers of birdwings puddling at 1600 hours and 0900–1600 hours. Data shown are the monthly means for six different months, with standard error bars.

The first of the four component studies to determine the sample requirements for counts of puddling birdwings assessed the optimum number of consecutive images needed to obtain a reliable count. Very little difference was found between the three sets of six five-minute block averages and their 95% confidence intervals for subsamples of one image or three images, or the full five images (Table 1). However, there was a very slight increase in average counts with increasing numbers of images. In the second component study, which aimed at determining the number of monitoring hours needed, counts at just a single hour, i.e. 1600 hrs, were seen to be positively and closely correlated with the average of hourly counts over an eight-hour period from 0900–1600 hrs (Pearson’s correlation coefficient, r = 0.986, P < 0.001) (Fig 3). And in the third study, which analyzed whether the numbers of birdwings puddling at a specific time of day could be predicted from counts made at an alternative time of day, the log transformed numbers of birdwings puddling at 1100 hrs (x) and 1600 hrs (y) were significantly and positively correlated (r^2^ = 0.973, P < 0.001) (S2 Fig), and the regression equation for prediction was y = 1.089x – 0.026. Thus, the number puddling at 1600 hrs could be estimated by the equation N_1_ = 10^(1.089×log(N2)– 0.026)^ where N_2_ is the number puddling at 1100 hrs. The fourth component study analyzed the number of monitoring days needed. In the correlation matrix for average counts on the first, second and third day at 1600 hrs, the Pearson correlation coefficients (r) were relatively high, but only one out of the three pairs of days was significantly correlated (Table 2).

**Table 1:** Comparison of the number of consecutive images needed to obtain a reliable puddling count. The figures show the averages and 95% confidence intervals of the number of puddling birdwings at two different times of the day for one, three and five images.

Number of images per 5-minute block	Time of day	
	1100–1130 hours	1500–1530 hours
1 image	95.3	102.7
	(83.7–107.0)	(92.1–113.2)
3 images	96.2	104.2
	(84.5–107.8)	(94.8–113.5)
5 images	97.0	105.1
	(86.0–108.0)	(96.4–113.9)

**Table 2:** Correlation matrix (r) for the different days of the monthly 1600 hrs birdwing counts.

	First day	Second day
**Second day**	0.943	
	(P = 0.005)	
**Third day**	0.722	0.831
	(P = 0.168)	(P = 0.081)

### Application of the technique

**Fig 4:**
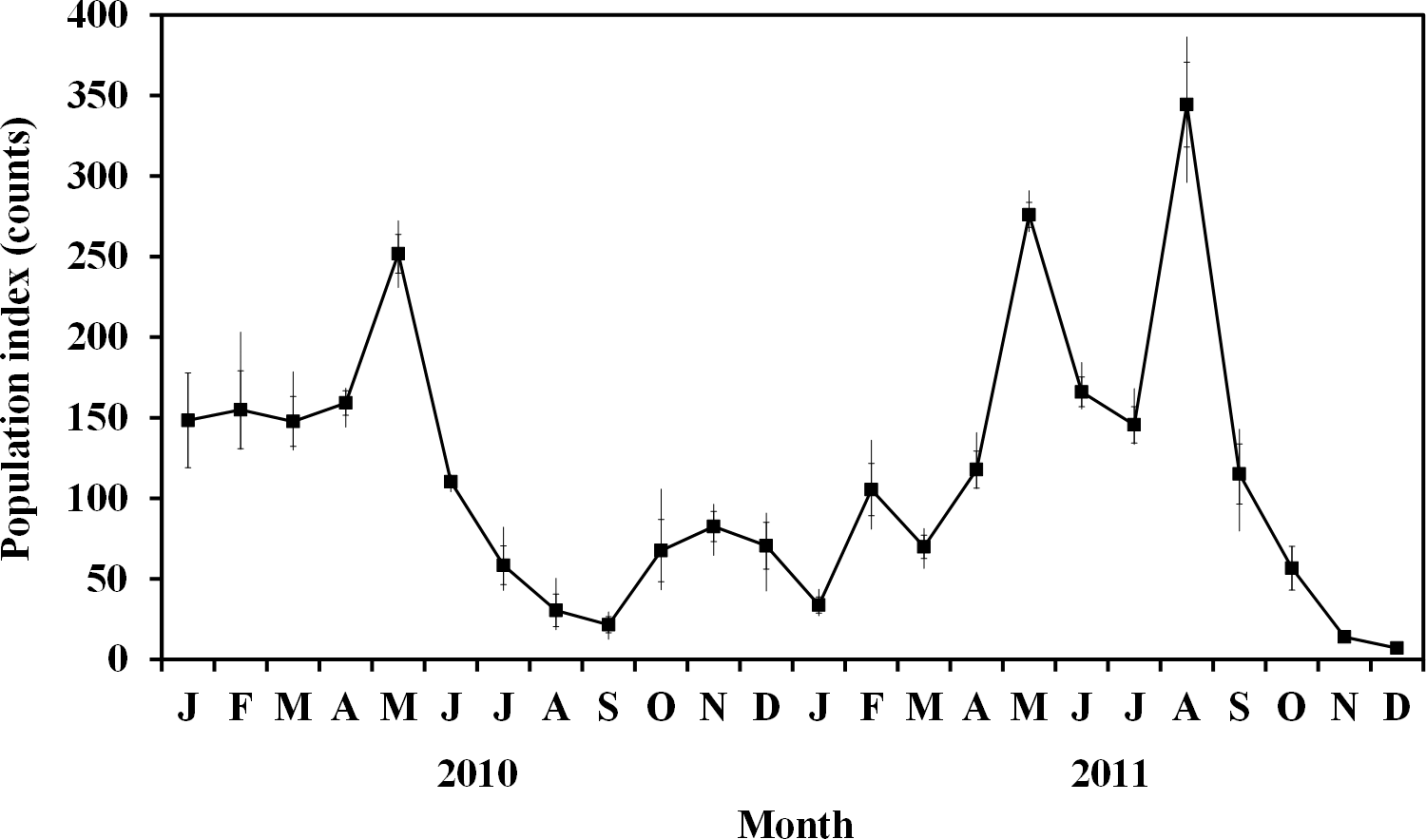
Fluctuation in the population index of monthly birdwing counts over a period of two years. Data shown are mean daily counts at puddles, the standard error and range for each month.

When puddling birdwings were monitored monthly for two years from 1400 to 1600 hrs for two to three consecutive days each month, a high degree of population fluctuation between months was observed. Average numbers ranged from only several puddling birdwings at low season to 344 at the peak. Variation between days in the month was small in comparison to the largest variations between months. The lowest population levels occurred in September of the first year and December of the second year. In the first year of population monitoring, two peaks occurred, the highest in May, followed by a much smaller peak in November (Fig 4). In the second year, there was a small peak in February followed by two much higher peaks in May and August.

In the analysis for the effects of environmental variables, there was a significant difference between monitoring months (F_22, 36_ = 41.28, P < 0.001) when controlling for the effect of the former. Birdwing counts also had a significant, positive association with relative humidity (F_1, 36_ = 6.84, P = 0.013; standardized regression coefficient, β = 0.270) (S1 Table). There was no significant association with brightness (F_1, 36_ = 3.91, P = 0.056, β = 0.160), temperature (F_1, 36_ = 0.70, P = 0.409, β = 0.128), rainfall (F_1, 36_ = 0.23, P = 0.636, β = 0.033), all-day rainfall (F_1, 36_ = 0.41, P = 0.524, β = -0.049) or all-day brightness (F_1, 36_ = 0.00, P = 0.980, β = -0.002) (S1 Table). When stepwise selection of variables was conducted at an α to enter or remove of 0.15, humidity and brightness were retained in the final model and showed a significant, positive association (P < 0.01) with birdwing counts (Table 3). Monitoring month (the first selected variable) remained significant (P < 0.001). The standardized coefficients for humidity and brightness were low, and hence also their relative effects on counts. The effect of humidity was slightly higher than the effect of brightness, and these variables were negatively and significantly correlated with each other (r = -0.785, P < 0.001) with a modest variance inflation factor (VIF = 1.85).

**Table 3:** General linear model for counts of puddling birdwings in response to monitoring month, relative humidity and brightness (final model).

Source	DF	Adj SS	Adj MS	F	P
Monitoring month	22	60.9967	2.7726	47.66	0.000
Relative humidity	1	0.4645	0.4645	7.98	0.007
Brightness	1	0.5797	0.5797	9.96	0.003
Error	40	2.3270	0.0582		
Total	64	64			
S = 0.2412; r^2^ = 96.36%; r^2^ (adjusted) = 94.18%; r^2^ (predicted) = 90.14%					
Regression term	Standardized Coefficient, β		SE of coefficient	T	P
Constant	-0.0424		0.0306	-1.39	0.173
Relative humidity	0.2017		0.0714	2.83	0.007
Brightness	0.1767		0.0560	3.16	0.003

## Discussion

### Comparison of transect counts and puddling counts

The significant, positive correlation between the numbers of birdwings puddling and numbers of birdwings in flight showed that the counts of puddling birdwings corresponded with the more traditional method of a transect count. Transect counts have been widely used in monitoring butterfly populations, for example in the studies of Van Swaay [29] and in the monitoring data available on PollardBase [30]. They have also been used in determining butterfly species diversity, for example by Basset et al. [31] and Majumder et al. [32]. Sometimes transect counts are used in conjunction with other methods such as fruit bait trapping, for example by Nganso et al. [33]. One of the best known transect counts is the Pollard walk [18] in which the recorder counts butterflies within a moving 5×5×5m box in a belt transect that may be divided into sections. The sum of the mean weekly count for the whole transect is used as a population index. Since the birdwings are not frequently seen in flight and are wide-ranging in their flight patterns, but can be recognized from a distance, we used a larger count zone that maximized counts, and at the same time made it possible to exclude recounts of the same birdwing circling the transect. Rarely was more than one birdwing seen in flight at any one time. Counts of birdwings puddling were much higher in magnitude than counts of birdwings in flight. The former would therefore be expected to be a more sensitive index of population size.

Like all methods of monitoring, puddling counts may be influenced to some degree by extraneous factors other than population size. Over a longer period of monitoring that examines trends over many years, these factors may be largely inconsequential to the interpretation of population trends. Variations in the availability of the resource over time and variations in attraction are the factors that could influence the counts of birdwings at the puddle. However, since the puddle is caused by a geothermal spring, it is stable year-round and unaffected by periods of dry weather. After rain, its geothermal and chemical properties are restored by underground seepage. The significant, positive correlation between the number of puddling birdwings counted on the ground and the number of patrolling birdwings counted in flight suggests that high and low numbers of puddling birdwings are not a result of temporal changes in puddling behavior such as changing levels of resource attraction, but rather proportional to variations in population size. Furthermore, there was no clear difference in the ratios of puddling to flying birdwings in the high- and low-season months, indicating that the aggregations were not obviously affected by group-size or conspecific visual attraction signals.

### Sample requirements for puddling counts

The first component study to determine the sample requirements for counts of puddling birdwings demonstrated that, in the absence of events that might disrupt puddling, just one image taken at each hour was sufficient to represent the numbers of birdwings puddling for the hour, because there was little difference between the averages and 95% confidence intervals when one, three or five images were used. The very slight increase in average counts with increasing numbers of images is to be expected, because the averages are centered one minute apart, and numbers of birdwings were on an increase during the 30-minute time periods on which the analyses were based. Where there is a disruptive event that causes the birdwings to take to flight, a delay of 10 minutes is sufficient to allow the birdwings to return before images are captured.

The strong positive correlation between average birdwing counts at 1600 hrs and the average of hourly counts over an eight-hour period (i.e., 0900–1600 hrs) in the second component study showed that just one hour’s sample at this peak puddling period was sufficiently representative of counts for the entire day. The results of the third study showed that the numbers of birdwings puddling during this peak hour of 1600 hrs could be predicted from the number of birdwings puddling at 1100 hours. Ideally, if a single, standardized hour is chosen for the monitoring of the birdwing at this site, it should be between 1400–1600 hours, which was observed to be the peak period for puddling activity. However, the flexibility to predict a standardized hour’s count from an alternative hour’s count is useful during seasons when heavy afternoon rain is expected. In the fourth component study, the lack of a consistent correlation between days indicated that there was a moderate amount of day to day variation in numbers of puddling birdwings within a month. This suggests that counts based on multiple days will yield a better estimate of the population than single-day counts.

### Application of the technique: Two-year monitoring

In the 24-month monitoring data, brightness and humidity at the time of monitoring had the most significant influence on day to day differences within a month, with higher numbers of birdwings when it was bright and humid. Bright sunshine usually resulted in lower humidity, as could be seen by the negative correlation between these two variables, but where brightness and humidity were both high, this would have enhanced puddling the most, although not greatly, as seen from the low standardized regression coefficients. On the other hand, the population showed great variation that was significant between months over the two-year period, with three peaks of about 250–350 puddling birdwings, and lows of less than 25 individuals. With such high monthly variation, the day to day differences due to daily weather patterns were relatively small. Variations in weather patterns and host plant phenology over the years could be responsible to some extent for the population fluctuation. There is no obvious wet or dry season in the geographical area in which the site is located, and weather patterns are not always consistent from one year to another. All-year-round monitoring is therefore preferable at this site.

One limitation of a population index based on puddling birdwings is that it excludes females. In subspecies *albescens*, however, males are more often seen than females due to their different habits. Wheeler’s [34] estimated based on field observations a ratio of one female to twenty males (quoted also by Straatman and Nieuwenhuis [35]). Eliot (in Corbet and Pendlebury [1]) considered this to be too high for the number of males. Sex ratio information from breeding experiments is not available for *T*. *b*. *albescens*. In subspecies *trogon* Vollenhoeven in Indonesia, the sex ratio was said to be approximately two males to one female among “several hundred” specimens bred by Straatman and Nieuwenhuis [35], but five males to four females among nine specimens bred by Dahelmi *et al*. [36]. The sex ratio of collected specimens of subspecies *mollumar*, which does not puddle in groups, was about equal [1]. It is uncertain if the observed high numbers of male *T*. *b*. *albescens* in comparison to females in the wild are a product only of differences in their behavior and hence differences in their detectability, or whether it is also reflective of an unequal sex ratio. The sex ratio of captive bred adults of *Parnassius apollo* (Linnaeus) in Poland was equal, but among naturally wild breeding adults and captive-bred adults that had been released, sex ratios were unequal and dissimilar from each other, with a stronger male bias in the naturally wild breeding adults [37]. This suggests that both perceived and real sex ratios in a field population can be different from the sex ratios of emerging adults, firstly due to behavioral differences in the sexes (and consequently differences in detectability) and secondly due to differential mortality rates between adults of different sexes. However, since the number of males in a butterfly population can be expected to correspond with the number of females (albeit by an unknown magnitude), monitoring based on an index of counts of males can be expected to be a reliable method of monitoring the overall population.

### Application of the technique: Developing a monitoring program

The success of a long-term monitoring program often depends on volunteers. Having a simple, rapid, practical and affordable monitoring protocol that requires the minimum effort to produce significant results encourages long-term volunteer involvement [38, 39]. Therefore, the current study focused not only on developing a reliable monitoring technique but also on simplification of the monitoring protocol. The method described here can be easily adopted by volunteers because of its simplicity. It is particularly suitable for monitoring the Rajah Brooke’s Birdwing population in Ulu Geroh because the village has a puddling site that is stable and attracts relatively large numbers of birdwings. Most of the puddling birdwings can be photographed at this site, and a small number outside this site can either be counted visually or omitted. Before monitoring starts, the puddles need to be observed for a short while to ensure that the birdwings are not experiencing a disturbance or recovering from one.

One image taken for each puddle at one representative hour would be sufficient as an indicator of the number puddling on a particular day. Three images taken at the start of the hour for three peak hours in a day is not difficult, and should be considered for better population index accuracy in the monitoring program. Having the counts repeated on different days, to average out daily variation due to weather conditions, is more important than replication within each day. For this reason, we would recommend that counts be conducted over three days, which is still very manageable, or at least two days if resources are truly limiting. The method also yields counts that are not excessively affected by small variations in weather. If it rains the whole day, counts can be conducted on a different day. When there is a high likelihood of afternoon rains, usually from the months of March to June, counts can be conducted in the morning and afternoon counts determined by prediction from the regression line.

### Adapting the method for other subspecies and species

The method can also be used to monitor populations of *albescens* in other areas, and can be used for another puddling subspecies, *brookiana*, which occurs in Borneo. A good understanding of the behavior of these birdwings is required at each site before implementing or adapting the method. For example, in Peninsular Malaysia’s Kuala Woh Recreational Forest, the birdwings puddle at scattered locations along the riverbanks and may move. In this case, all the puddling birdwings should be sampled rather than those at fixed puddling locations, but they should be within a fixed belt transect of sufficient length to cover as many puddling sites as possible. In areas such as Kenaboi Forest Reserve where the birdwings rarely puddle but are commonly seen in flight at specific locations, a transect walk count or single-point observation count should be used. A similar count strategy should be used for subspecies *mollumar* in the east of Peninsular Malaysia (sometimes referred to as ssp. *trogon*), which is rarely seen puddling, but the transects need to be much longer, as sightings of this subspecies are much less frequent.

We envisage that the method can also be adapted to monitor other puddling butterfly species, including mixed species groups. While camera trapping is widely used to monitor larger wildlife [40], and night photography has even been used to monitor congregating fireflies in Malaysia [41], photography of puddling butterflies has yet to be used as a method for monitoring butterflies perhaps partly because of the difficulty of counting large numbers of puddling butterflies and partly because they are viewed as unstable aggregations. However, advances in digital camera technology have made it possible to count large numbers of puddling butterflies with relatively little cost, and the aggregations of *T*. *brookiana* in Ulu Geroh are highly stable. In other puddling groups of butterflies, if the puddles are sampled over a sufficiently long transect and include some stable puddling sites such as may occur at seepages and the edges of streams and rivers, it should provide a stable enough count, provided puddles that unnaturally increase counts in the short term, for example because of urine or garbage, are excluded. Preliminary tests similar to those conducted in this study need to be carried out before the method can be used for monitoring mixed puddling groups. The species identification of some puddling butterflies in highly diverse regions may be difficult even in digital images taken at puddles because of the small differences that separate the species and because other puddling butterflies may obscure parts of their wings. In such species, identification to the generic level may be all that is possible, just as in other techniques such as observational transect counts where there is no actual capture and handling of the butterflies. However, many butterfly species can be readily identified to species level in images of puddling butterflies. Examples in Peninsular Malaysia include species of distinctive color (e.g., *Appias nero* (Fabricius)), wing shape (e.g., *Saletara liberia* (Cramer)) or pattern (e.g., *Graphium sarpedon* (Linnaeus)). Among those that may be difficult to identify to species level are some species of *Eurema* (Pieridae) and *Graphium* (Papilionidae) and some small species of Polyommatinae (Lycaenidae) that commonly puddle.

## Supporting information

S1 Fig Numbers of birdwings puddling at minute-intervals before and after a disruptive event. (TIF)

S2 Fig Regression of numbers of birdwings puddling at 1600 against numbers puddling at 1100 hrs. Data shown are the monthly means and standard errors (data on both axes log transformed). The x-axis is taken as an independent variable for the purpose of obtaining a prediction equation.
(TIF)

S1 Table General linear model for counts of puddling birdwing in response to all variables (full model). (DOCX)
