# Correction: Monitoring butterflies using counts of puddling males: A case study of the Rajah Brooke’s Birdwing (*Trogonoptera brookiana albescens*)

**DOI:** 10.1371/journal.pone.0196304

**Published:** 2018-04-18

**Authors:** The PLOS ONE Staff

In the Author Contributions section, it should be noted that Norma-Rashid Yusoff also contributed to Supervision.

The Funding section is incomplete. The complete funding information is: This work was supported by Malaysian Forest Research and Development Board (Grant number: GPP-0609-FA-02) received by CK; Postgraduate Research Fund from University of Malaya (Grant number: PV131/2012A) received by NY; Malaysian Economic Planning Unit and Ministry of Natural Resources and Environment, Malaysia. The funders had no role in study design, data collection and analysis, decision to publish, or preparation of the manuscript.

The abbreviation for the third author is incorrect in the citation. The correct citation is: Phon C-K, Kirton LG, Norma-Rashid, Y (2017) Monitoring butterflies using counts of puddling males: A case study of the Rajah Brooke’s Birdwing (*Trogonoptera brookiana albescens*). PLoS ONE 12(12): e0189450. https://doi.org/10.1371/journal.pone.0189450

There is an error in the URL link for Reference 9. The correct reference is: Baioumy HM, Nawawi M, Wagner K, Arifin MH. Geological setting and origin of non-volcanic hot springs in West Malaysia. GEOS 2014: Proceedings of the 3rd International Conference on Geological and Earth Sciences; Singapore. 2014. pp. 14–18. doi:10.5176/2251-3353_GEOS14.37.

The publisher apologizes for these errors.
