# Supplementary figures and images for: Monitoring butterflies using counts of puddling males: A case study of the Rajah Brooke's Birdwing (Trogonoptera brookiana albescens)
Source: PLoS One. 2017 Dec 12;12(12):e0189450. doi: 10.1371/journal.pone.0189450 (PMC5726648; doi:10.1371/journal.pone.0189450)

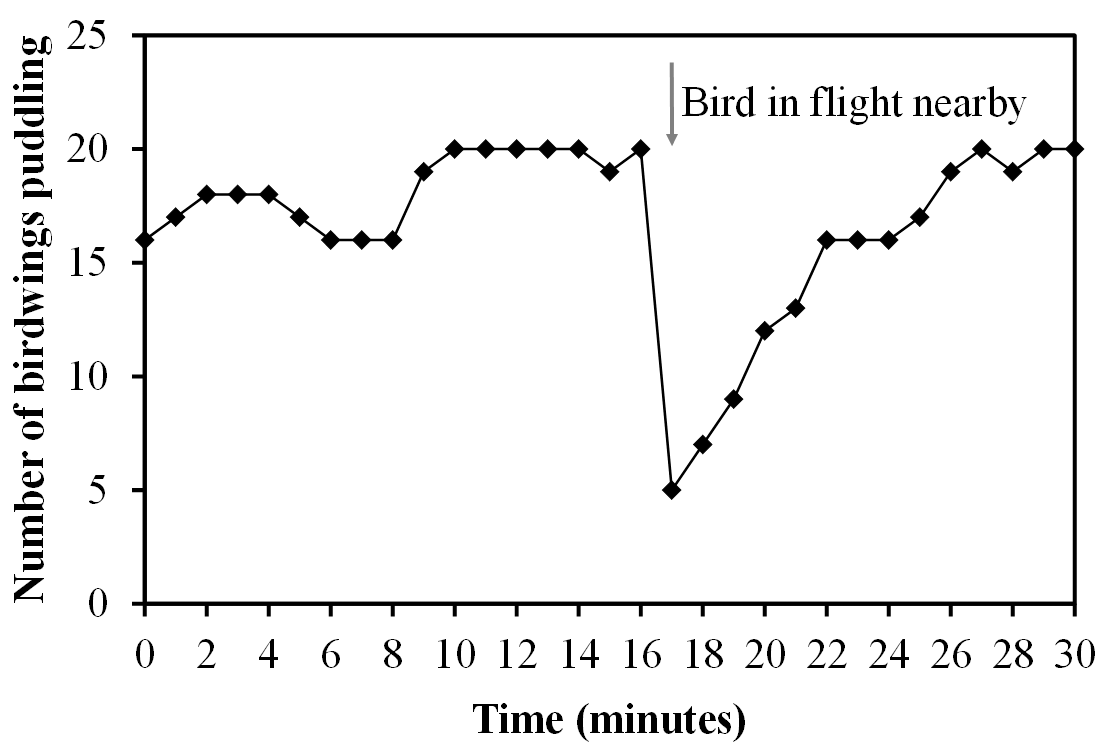

Supplement: S1 Fig — (TIF) [file pone.0189450.s001.tif]

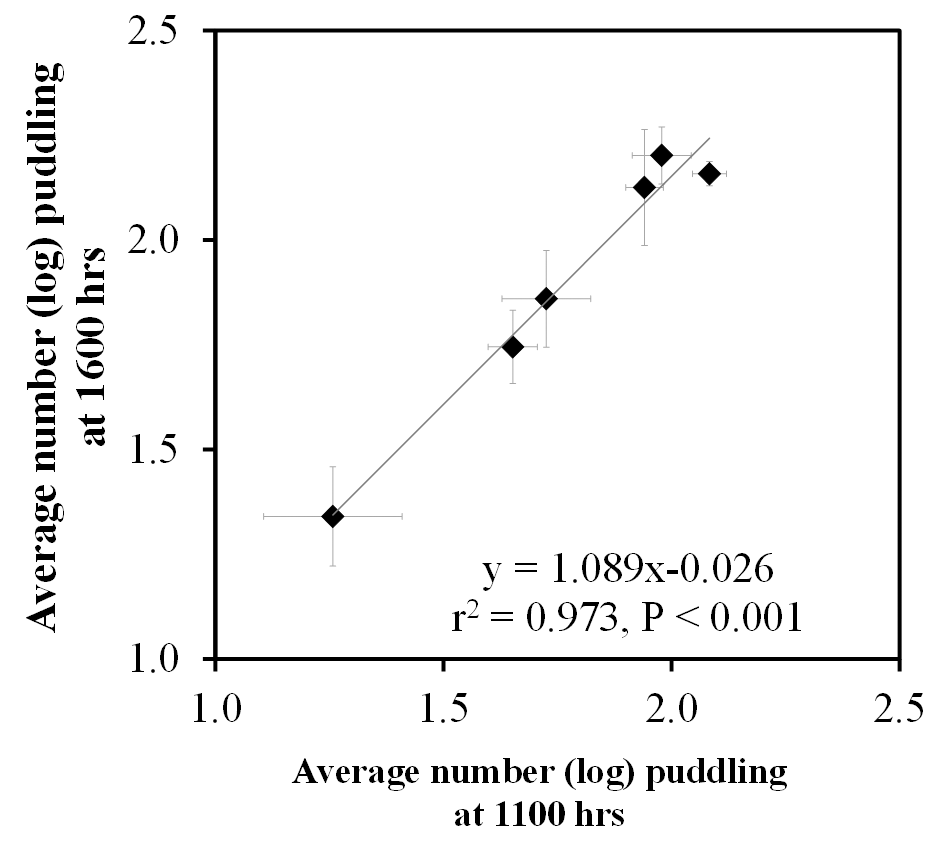

Supplement: S2 Fig — Data shown are the monthly means and standard errors (data on both axes log transformed). The x-axis is taken as an independent variable for the purpose of obtaining a prediction equation. (TIF) [file pone.0189450.s002.tif]
