# Supplementary material for: Monitoring butterflies using counts of puddling males: A case study of the Rajah Brooke's Birdwing (Trogonoptera brookiana albescens)
Source: PLoS One. 2017 Dec 12;12(12):e0189450. doi: 10.1371/journal.pone.0189450 (PMC5726648; doi:10.1371/journal.pone.0189450)
Supplement: S1 Table — (DOCX) [file pone.0189450.s003.docx]

**General linear model for counts of puddling birdwing in response to all variables (full model).**

| Source | DF | Adj SS | | Adj MS | F | P |
| --- | --- | --- | --- | --- | --- | --- |
| Monitoring month | 22 | 56.1522 | | 2.5524 | 41.28 | 0.000 |
| Relative humidity | 1 | 0.4232 | | 0.4232 | 6.84 | 0.013 |
| Brightness | 1 | 0.2417 | | 0.2417 | 3.91 | 0.056 |
| Temperature | 1 | 0.0432 | | 0.0432 | 0.70 | 0.409 |
| All-day rainfall | 1 | 0.0255 | | 0.0256 | 0.41 | 0.524 |
| Rainfall | 1 | 0.0141 | | 0.0141 | 0.23 | 0.636 |
| All-day brightness | 1 | 0.0000 | | 0.0000 | 0.00 | 0.980 |
| Error | 36 | 2.2257 | | 0.0618 |  |  |
| Total | 64 | 64 | |  |  |  |
| S = 0.2486; r^2^ = 96.52%; r^2^ (adjusted) = 93.82%; r^2^ (predicted) = 88.29% | | | | | | |
| Regression term | Standardized Coefficient, β | | SE of coefficient | | T | P |
| Constant | -0.039 | | 0.032 | | -1.22 | 0.232 |
| Relative humidity | 0.270 | | 0.103 | | 2.62 | 0.013 |
| Brightness | 0.160 | | 0.081 | | 1.98 | 0.056 |
| Temperature | 0.128 | | 0.153 | | 0.84 | 0.409 |
| All-day rainfall | -0.049 | | 0.077 | | -0.64 | 0.524 |
| Rainfall | 0.033 | | 0.069 | | 0.48 | 0.636 |
| All-day brightness | -0.002 | | 0.085 | | -0.03 | 0.980 |
